# Cardiovascular Outcomes Associated With Exposure To Radiation Therapy In Thoracic Malignancies: An Insight Study Using the National Inpatient Database

**DOI:** 10.7759/cureus.47113

**Published:** 2023-10-16

**Authors:** Mahir Yilmaz, Ekrem Turk, Muhammad K Sana, Ayobami Olafimihan, Ibrahim Uygun, Sami Shoura, Kumar K Batra

**Affiliations:** 1 Internal Medicine, John H. Stroger, Jr. Hospital of Cook County, Chicago, USA; 2 Hematology-Oncology, John H. Stroger, Jr. Hospital of Cook County, Chicago, USA

**Keywords:** valvular heart disease, pericardial diseases, conduction disorders, cardiomyopathy, coronary artery disease, congestive heart failure, chest radiation, thoracic malignancy, radiation therapy, cardiovascular outcomes

## Abstract

Background

Thoracic irradiation is a widely used therapeutic and palliative treatment option for thoracic neoplasms. However, short- and long-term cardiovascular adverse effects of radiation exposure remain a major concern. The short-term adverse effects are observed within months of exposure such as pericardial diseases; meanwhile, the long-term complications are usually insidious and manifest over decades, such as congestive heart failure, coronary artery disease, cardiomyopathy, conduction disorders, constrictive pericarditis, and valvular heart disease. Hence, long-term cardiovascular adverse effects are challenging to predict, and the association with radiation exposure remains difficult to establish.

Methodology

This retrospective, observational study was conducted using data from the National Inpatient Sample (NIS) database from 2016 to 2019. Adult patients with primary thoracic malignancies who underwent radiation therapy (RT) were defined using principal and secondary International Classification of Diseases, Tenth Revision codes. Other malignancies that can be treated with RT and all secondary malignancies were excluded from the primary comparison group. Cardiac outcomes were defined as the prevalence of congestive heart failure, coronary artery disease, cardiomyopathy, conduction disorders, pericardial diseases, and valvular heart diseases in the primary group. The multivariate logistic and the linear regression analyses were used to adjust for confounders.

Results

When compared to the general population, adults with thoracic malignancies exposed to RT had higher odds of developing chronic pericarditis (adjusted odds ratio (aOR) = 2, 95% confidence interval (CI) = 1.9-2.2, p < 0.001), acute pericarditis (aOR = 2.3, 95% CI = 1.9-2.9, p < 0.001), constrictive pericarditis (aOR = 2.8, 95% CI = 2.1-3.7, p < 0.001), conduction disorders (aOR = 1.3, 95% CI = 1.2-1.35, p < 0.001), coronary artery disease (aOR = 1.24, 95% CI = 1.2-1.27, p < 0.001), heart failure (aOR = 1.44, 95% CI = 1.4-1.5, p < 0.001), and valvular heart disease (aOR = 1.37, 95% CI = 1.3-1.4, p < 0.001). There was no difference in the odds of developing cardiac arrest (aOR = 1, 95% CI = 0.9-1.10, p = 0.6) or acute myocardial infarction (aOR = 1.1, 95% CI = 1-1.15, p < 0.001). When compared to adults with thoracic malignancies not exposed to RT, adults with thoracic malignancies who were exposed to RT had higher odds of developing acute myocardial infarction (aOR = 1.14, 95% CI = 1.1-1.18, p < 0.001), chronic pericarditis (aOR = 1.3, 95% CI = 1.2-1.3, p < 0.001), acute pericarditis (aOR = 1.6, 95% CI = 1.2-2.1, p < 0.001), constrictive pericarditis (aOR = 2.2, 95% CI = 1.5-3.2, p < 0.001), conduction disorders (aOR = 1.1, 95% CI = 1.08-1.13, p < 0.001), coronary artery disease (aOR = 1.14, 95% CI = 1.12-1.16, p < 0.001), heart failure (aOR = 1.2, 95% CI = 1.17-1.23, p < 0.001), and valvular heart disease (aOR = 1.3, 95% CI = 1.2-1.35, p < 0.001). The odds were similar between the two groups for developing cardiac arrest (aOR = 0.86, 95% CI = 0.8-0.98, p = 0.05).

Conclusions

Adults with thoracic malignancies who were treated with RT have higher odds of developing chronic pericarditis, acute pericarditis, constrictive pericarditis, conduction disorders, coronary artery disease, heart failure, and valvular heart disease while similar odds of developing cardiac arrest or acute myocardial infarction compared to the general adult population.

## Introduction

Radiation therapy (RT) has been widely utilized to treat or palliate many types of cancer effectively for more than a century and has become readily available. Every other patient with malignancy is exposed to radiation at some point during the course of treatment [1]. RT uses high-energy X-rays or other particles to destroy both cancer and healthy cells, as a result of which patients may suffer from short-term as well as long-term adverse effects. Chest RT is commonly employed to treat breast, lung, and esophageal solid malignancies and lymphomas. RT has been associated with improved local control and survival rates, underscoring its contribution to the armamentarium of cancer treatment. However, despite vast expertise and its numerous benefits, chest RT is associated with unintended adverse effects on healthy tissues, particularly associated with the heart, which remain a diagnostic challenge to date.

Radiation-induced heart disease (RIHD) is a spectrum of heart diseases, including pericarditis, cardiomyopathy, coronary artery disease (CAD), valvular heart disease, and cardiac conduction abnormalities, usually manifesting nearly 20 years after radiation exposure [2]. A Japanese study on atomic bomb survivors in Hiroshima and Nagasaki showed a higher risk of heart disease even at lower doses after 50 years of follow-up [3]. A recent study on breast cancer patients showed that chest RT was associated with a 1.7-fold (95% confidence interval (CI) = 1.2-2.5) increase in mortality from cardiovascular disease compared to surgery after an average of 14 years of follow-up [4]. Another study using stress echocardiography and myocardial perfusion imaging found a 2.7% prevalence of severe, three-vessel, or left main CAD and a 7.5% prevalence of coronary stenosis greater than 50% in patients treated with mediastinal irradiation at an average of 15 years of follow-up [5].

Multiple mechanisms of RIHD have been postulated before, including direct injury to cardiac tissues by radiation, localized inflammatory response eventually leading to fibrotic remodeling, generation of reactive oxygen species causing oxidative stress, and damage to the vascular endothelium promoting a pro-thrombotic and pro-inflammatory environment [6].

CAD is the most common manifestation of RIHD, with an incidence of 50-80%, resulting in angina, myocardial infarction, and even sudden cardiac death [1]. Fibrotic changes in the heart valves can result in valvular stenosis or regurgitation, with aortic regurgitation being the most common valve problem in RIHD [7]. Thoracic radiation affects the left-sided valves more than the right side, given higher systemic pressures and the aortic valve being closest to the chest wall. The pericardial disease of RIHD has varying manifestations based on the duration of exposure, with acute pericarditis and cardiac tamponade being prominent short-term complications, especially after a high irradiation dose. Chronic pericarditis and constrictive pericardial disease are long-term complications due to repetitive inflammation and fibrosis. In pericardium, asymptomatic fibrin and collagen deposition is seen in nearly 70% of patients with thoracic irradiation history [8]. Arrhythmias, sinus node dysfunction, atrioventricular blocks, and QT prolongation are typical conduction problems after mediastinal RT and are anticipated to be seen in the short term. Fibrosis in cardiac conduction pathways and myocardial ischemia alters the electrical physiology of the heart, and conduction abnormalities are seen in about 5% of RIHDs [9]. Pre-existing CAD and other known cardiac risk factors such as hypertension, dyslipidemia, diabetes, family history of CAD, active smoking, and sedentary lifestyle further increase the risk of RIHD [10].

Various patient-specific and treatment-related factors also influence the risk of developing RIHD. These include the cumulative radiation dose, radiation field, fractionation schedule, exposure age, and pre-existing cardiovascular risk factors. Modifying these factors and employing advanced RT techniques and cardioprotective medications can help minimize the risk of RIHD while ensuring optimal oncologic outcomes.

## Materials and methods

Study design

This retrospective cohort study used the National Inpatient Sample (NIS) database from 2016 to 2019. The NIS is the largest publicly available inpatient database in the United States (US), which is a part of the Healthcare Quality and Utilization Project, sponsored by the Agency for Healthcare Research and Quality. The NIS contains discharge data from a 20% stratified sample of all discharges from US community hospitals, excluding rehabilitation and long-term acute care hospitals. It was designed as a stratified probability sample representing all non-federal acute-care hospitals nationwide. Hospitals are stratified based on ownership, urban or rural area, geographic region, bed size, and teaching status. All discharges from these hospitals are weighted to be nationally representative and cover more than 97% of the US population.

In the NIS, The International Classification of Diseases, Tenth Revision, Clinical Modification/Procedure Coding System (ICD-10-CM/PCS) is used for coding. The diagnoses are divided into the following two categories: principal and secondary. A principal diagnosis was the main ICD-10 code for hospitalization, and secondary diagnoses were any ICD-10 code other than the principal diagnosis. Unfortunately, there is no reliable mechanism to differentiate secondary diagnoses coded during the index hospital admission from those coded in the previous admissions.

As all patient data in the NIS are de-identified and publicly available, our study was exempt from ethical review.

Patient population

Our initial population (thoracic malignancies) was extracted from the 2016-2019 NIS database, pinpointing patients with primary or secondary diagnosis codes corresponding to various thoracic cancers. Specific ICD-10 codes used for identification are as follows: primary lung cancers and mesotheliomas: C34, C399, Z8511, D022, and C450; Hodgkin lymphomas: C81 and Z8571; breast cancers: C50, Z853, and D05; esophageal cancer: C15, Z8501, and D001; thymic cancers: C37 and Z8523; and mediastinal cancers: C761 and C38.

Inclusion and exclusion criteria

**Figure 1:**
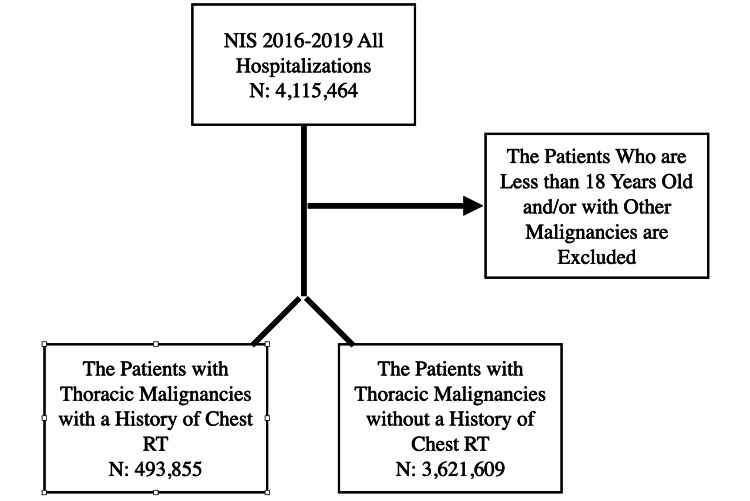
Study design. NIS = National Inpatient Sample; RT = radiation therapy

The thoracic malignancies group was then stratified into the following two groups: patients with thoracic malignancies and a history of chest RT and those without RT. Patients younger than 18 and with any other coding for cancers other than those defined above or for any metastasis were excluded from our initial thoracic malignancies group (Figure 1).

For every admission, data on patient demographic characteristics of age, sex, and race; primary payer information; median household income; and hospital characteristics were extracted. Relevant medical comorbidities were also defined.

Outcomes

The primary outcomes were odds of developing cardiac arrest, acute decompensated heart failure, congestive heart failure, systolic heart failure, diastolic heart failure, coronary artery disease, acute myocardial infarction, cardiomyopathy, conduction disorders, acute pericarditis, constrictive pericarditis, chronic pericarditis, cardiac tamponade, valvular heart disease, aortic stenosis, and aortic insufficiency in the case cohort.

The secondary outcomes were in-hospital mortality, the length of stay, and the total hospital charges of hospitalized patients for the primary diagnosis of acute decompensated heart failure in the case cohort.

Statistical analysis

The STATA version 17 (StataCorp, TX, USA) was used to analyze the data. Continuous and categorical variables were described as means and percentages, respectively. Differences in baseline characteristics were compared using a chi-square test for categorical variables and a Student’s t-test for continuous variables.

Variables from the relevant literature search and medical comorbidities in the result tables were tested using univariate logistic regression analysis to calculate unadjusted odds ratios for the primary outcome. The final multivariate regression model included variables with p-values <0.1 from the initial univariate screen.

The Charlson Comorbidity Index (CCI), recorded in the NIS database, was also used to adjust for the comorbidity burden. The CCI is a summary comorbidity measure and was used as a potential confounder in the multivariate regression analysis, given comparable performance to each component of CCI used separately.

The multivariate logistic and linear regression model, with all variables and comorbidities, were used to adjust for confounders accordingly. We presented both the crude and adjusted values for these outcomes in this study.

## Results

Baseline characteristics

The query of NIS from 2016 to 2019 revealed a substantial group of 4,115,464 adult hospitalizations involving patients with primary thoracic malignancies. These admissions encompass adults who were either admitted with a primary diagnosis of malignancy or had a history of malignancy alongside another reason for admission. Within this cohort, a total of 493,855 individuals with thoracic malignancies underwent RT (cases), accounting for 12% of all primary thoracic malignancies identified in the study. The majority of patients in the case cohort consisted mainly of individuals with breast cancer (58%), followed by lung cancer (35%). The case cohort had a mean age of 68, which was, on average, younger than that in the control group (mean age = 70, p < 0.001). Despite the difference in age, the gender distribution remained consistent across both groups, with a female predominance among cases and controls ((76% vs. 77%, respectively). The predominant racial composition among cases and controls consisted of individuals of white race (74% and 75%, respectively). Moreover, a statistically significant higher proportion of black patients was observed in cases compared to controls (13% vs. 11.9%, p < 0.001). This underscores the importance of underutilization of RT among the black population with thoracic malignancies.

The evaluation of overall health status using the CCI revealed a similar mean score for cases and controls, with scores of 3 and 3.05, respectively, indicating similar distribution of comorbidity burden between the two cohorts. The distribution of major comorbidities was similar in cases and controls, including hypertension (42% vs. 43%, respectively) and hyperlipidemia (43.9% vs. 43.2%, respectively) were observed most commonly. However, a notable difference was found in the prevalence of smoking history, with cases exhibiting a significantly higher occurrence (41%) compared to controls (33%) with a p-value of less than 0.001.

Interestingly, our data also revealed that controls had a higher incidence of chemotherapy usage compared to cases (57% vs. 50%, p < 0.001). This could potentially indicate a preferential use of chemotherapy over radiation in certain patient subsets, which necessitates further investigation into the decision-making processes regarding the selection of therapeutic modality in thoracic malignancies.

Table 1 provides a detailed overview of the remaining descriptive data and comorbid conditions across the two groups. Further discussion and analysis of these findings will be done in the subsequent sections of this manuscript.

**Table 1:** Baseline characteristics and distribution of comorbidities between adults with thoracic malignancies who received radiation therapy (cases) versus those who did not receive radiation therapy (controls). The last column shows the total population group that involves adult patients with thoracic malignancies regardless of the chest radiation therapy status.

	Cases	Controls	P-value	Total
Total sample size (%)	493,855 (12%)	3,621,609 (88%)	N/A	4,115,464
Distribution of malignancies				
Lung cancer	34.0%	35.0%	N/A	N/A
Hodgkin lymphoma	3.8%	3.0%	N/A	N/A
Breast cancer	57%	58%	N/A	N/A
Esophageal cancer	7.7%	4.0%	N/A	N/A
Mediastinal cancer	0.1%	0.3%	N/A	N/A
Thymic cancer	0.3%	0.2%	N/A	N/A
Age (%)				
Mean age	68	70	N/A	70
18-40	2.0%	2.2%	<0.001	3%
40-60	20.0%	16.0%	<0.001	17%
60-80	58.0%	54.0%	<0.001	54%
>80	20.0%	27.8%	<0.001	26%
Gender (%)				
Female	76.0%	77.0%	<0.001	77.6%
Race				
White	74.0%	75.0%	0.02	74.9%
Black	13.0%	11.9%	<0.001	12%
Latin	6.0%	5.7%	<0.001	5.7%
Other	7.0%	7.4%	<0.001	7.2%
Insurance				
Medicare	68.5%	72.5%	<0.001	70.5%
Medicaid	7.5%	7.5%	0.06	7.2%
Private insurance	22.8%	19.0%	<0.001	19%
Self-pay	1.0%	1.2%	<0.001	1.1%
Median household income				
1-24,999$	24.5%	27.0%	<0.001	27%
25,000-34,999$	24.5%	25.5%	<0.001	25.5%
35,000-44,999$	25.0%	24.5%	<0.001	25%
>45,000$	25.0%	22.0%	<0.001	22.5%
Hospital region				
Northeast	21.0%	20.0%	<0.001	20.7%
Midwest	28.0%	24.0%	<0.001	24%
South	33.0%	39.0%	<0.001	38%
West	18.0%	17.0%	<0.001	17%
Hospital size				
Small	19.0%	20.0%	<0.001	20%
Medium	27.0%	30.0%	<0.001	29%
Large	54.0%	50.0%	<0.001	51%
Hospital location				
Rural	5.0%	8.0%	<0.001	9%
Urban	95.0%	92.0%	<0.001	91%
Distribution of comorbidities				
Diabetes	25.0%	27.0%	<0.001	27%
Hypertension	42.0%	43.0%	<0.001	43.5%
Hyperlipidemia	43.9%	43.2%	<0.001	43%
Obesity	13.0%	12.0%	<0.001	12.6%
Smoking	41.0%	33.0%	<0.001	34%
History of chemotherapy	50.0%	5.7%	<0.001	11%
Charlson Comorbidity Index				
Mean	3	3.05	N/A	3
Category 0	53.0%	48.0%	<0.001	49%
Category 1	33.0%	35.0%	<0.001	35%
Category 2	10.0%	12.0%	<0.001	11%
Category 3	4.0%	5.0%	<0.001	5%

Primary and secondary outcomes

In our comprehensive analysis of the NIS database, we aimed to explore the association between RT for thoracic malignancies and the risk of developing cardiac disorders compared to thoracic malignancies without RT. Our findings present compelling evidence indicating a higher prevalence of multiple cardiac conditions in patients with a history of RT for thoracic malignancies (Table 2).

**Table 2:** Cardiovascular outcomes in adults with thoracic malignancies who received radiation therapy (cases) versus no radiation therapy (controls).

	Cases	Controls	Odds ratio	P-value	95% confidence interval
Cardiac arrest	0.6%	0.7%	0.86	0.05	0.78-0.95
Coronary artery disease	25.6%	25.7%	1.14	<0.001	1.12-1.16
Acute myocardial infarction	4.9%	4.7%	1.14	<0.001	1.10-1.18
Acute decompensated heart failure	9.2%	8.6%	1.2	<0.001	1.17-2.4
Valvular heart disease	8.8%	7.2%	1.3	<0.001	1.2-1.35
Aortic stenosis	2.8%	2.3%	1.39	<0.001	1.3-1.47
Aortic insufficiency	0.6%	0.4%	1.3	<0.001	1.2-1.5
Diastolic heart failure	11.9%	11.7%	1.2	<0.001	1.1-1.25
Systolic heart failure	8.4%	7.4%	1.17	<0.001	1.1-1.2
All heart failure	21.9%	21.7%	1.2	<0.001	1.17-1.23
All cardiomyopathies	5.0%	4%	1.29	<0.001	1.1-1.37
Constrictive pericarditis	Negligible	Negligible	2.2	<0.001	1.5-3.2
Acute pericarditis	0.1%	Negligible	1.6	<0.001	1.2-2.1
Chronic pericarditis	1.8%	1.2%	1.3	<0.001	1.2-1.3
All conduction problems	27.6%	27.5%	1.1	<0.001	1.08-1.13

Specifically, we found a statistically significant increased risk for acute myocardial infarction in patients who received RT, with an adjusted odds ratio (aOR) of 1.14 (95% CI = 1.1-1.18, p < 0.001). This indicates that these patients were 14% more likely to develop acute myocardial infarction compared to controls. A similar trend was observed for chronic pericarditis with an aOR of 1.3 (95% CI = 1.2-1.3, p < 0.001), implying a 30% higher likelihood of developing this condition following RT. Furthermore, the risk of acute pericarditis was considerably higher, with an aOR of 1.6 (95% CI = 1.2-2.1, p < 0.001), showing a 60% increased likelihood of occurrence.

Our analysis further revealed a strong association between RT and constrictive pericarditis. The aOR of 2.2 (95% CI = 1.5-3.2, p < 0.001) represents a two-fold risk of constrictive pericarditis. The case cohort demonstrated a higher prevalence of conduction disorders and CAD, with aORs of 1.1 (95% CI = 1.08-1.13, p < 0.001) and 1.14 (95% CI = 1.12-1.16, p < 0.001), respectively. These findings further highlight the higher cardiovascular mortality in the case cohort, suggesting a 10-14% increased risk for these conditions. Similarly, heart failure and valvular heart disease exhibited a higher prevalence of cases with aORs of 1.2 (95% CI = 1.17-1.23, p < 0.001) and 1.3 (95% CI = 1.2-1.35, p < 0.001), respectively. The analysis revealed similar odds of developing cardiac arrest between the two groups with an aOR of 0.86 (95% = CI 0.8-0.98, p = 0.05).

These findings provide valuable insights into the cardiac outcomes associated with RT for thoracic malignancies. This knowledge can assist clinicians in making informed decisions and implementing proactive strategies to effectively manage and minimize cardiac risks in patients undergoing such treatment.

## Discussion

This analysis sheds light on the prevalence of cardiac outcomes associated with radiation exposure in thoracic malignancies compared to those with no exposure. The current literature is largely observational based on long-term follow-ups of patients who were irradiated three decades ago [11]. Our analysis has several implications in clinical practice with the latest prevalence and associations from the NIS, which is the largest database of the US population from 2016 to 2019. These findings echo the results of previous studies and build upon the current body of literature on this critical issue and the continuous need for surveillance and monitoring.

In our analysis, the observed association between RT and an elevated risk of acute myocardial infarction aligns with earlier work conducted by Darby et al. They identified a relationship between radiotherapy and CAD, culminating in acute myocardial infarction, particularly among patients with left-sided breast cancer [12].

Our study also indicated an elevated prevalence of chronic and acute pericarditis among patients who underwent RT, reaffirming prior findings regarding potential pericardial complications of thoracic irradiation [13]. Furthermore, the greater prevalence of constrictive pericarditis in our study substantiates the work of Bradley et al., who identified radiation-induced pericarditis as a well-established consequence of RT [14].

The link between RT and conduction disorders, as well as CAD, discovered in our study has also been emphasized in previous research. Cutter et al., for instance, highlighted a connection between RT for thoracic malignancies and conduction abnormalities, contributing to long-term cardiac morbidity [15].

Our results regarding the relationship between RT and increased prevalence of heart failure valvular heart disease are in accordance with existing evidence. Gujral et al. demonstrated that radiation can induce valvular damage, leading to such conditions [16].

Interestingly, we did not find a notable increase in the risk of cardiac arrest in patients with a history of RT. This result, seemingly contrary to our other findings, aligns with other studies suggesting that while RT increases the risk of chronic cardiac conditions, it does not directly lead to a higher risk of acute events such as cardiac arrest [17].

These findings underscore the importance of rigorous cardiovascular assessments and risk management strategies in patients with thoracic malignancies who have received or are about to receive RT. The increased risk of various cardiac conditions necessitates a multidisciplinary approach, wherein oncologists and cardiologists collaborate to optimize patient outcomes.

International Cardio-Oncology Society expert consensus statement 2021 supports the baseline cardiovascular assessment with history, physical examination, electrocardiogram, and transthoracic echocardiogram in patients planned to receive RT with repeat transthoracic echocardiograms in high-risk asymptomatic subset at 6-12 months starting after one year of RT and ischemic evaluation after five years. Baseline coronary artery calcification assessment should be reviewed if available but is not recommended to be obtained solely for the purpose of surveillance as it is recommended not to change the RT regimen based on the degree of calcification [18].

Similarly, a joint consensus statement was published by the American Society of Echocardiography and the European Association of Cardiovascular Imaging in 2013, which also emphasizes the need for annual screening with history and physical examination. The consensus also supports echocardiography after five years of exposure in high-risk patients with an alternative non-invasive stress test [19].

Further research is warranted to understand the mechanisms underlying RT-induced cardiac diseases. Such understanding could potentially lead to innovative strategies for risk mitigation, early detection, and treatment of these conditions. Our findings also stress the importance of considering these risks when choosing between therapeutic modalities for patients with thoracic malignancies.

The need for vigilant monitoring and proactive risk management in this patient group is evident from our findings. The implementation of individualized care plans that take into consideration the patient’s potential comorbidities, lifestyle factors, and age could help improve the management of patients who receive RT [20].

Though there have been several mechanisms of pathophysiology proposed in the literature, the exact mechanisms remain obscure. Further investigations into the precise pathophysiological mechanisms of radiation-induced cardiac damage are required. This could provide insights into potential preventive strategies and guide the development of novel treatment options [21].

The findings of our study highlight the need for a collaborative approach among oncologists, cardiologists, and RT in managing patients with thoracic malignancies. This reinforces the importance of developing comprehensive treatment plans that account for the patient’s complete health profile, including potential cardiac risks [22]. Despite the advances in RT technology, the exposure is persistently associated with high cardiovascular comorbidity. As the effect of radiation exposure is insidious in onset and takes several decades to unmask, we may not yet have seen the outcomes of advances and modifications in RT.

## Conclusions

Despite advancements in RT and surveillance, radiation exposure in thoracic malignancies continues to be associated with a heightened risk of cardiovascular complications. Our findings, derived from the comprehensive analysis of the NIS database, highlight a persistently elevated risk for various cardiac conditions in patients treated with RT for thoracic malignancies. The diverse cardiac morbidities include acute myocardial infarction, pericarditis, conduction disorders, CAD, and heart failure, among others. While the association with cardiac arrest was not significant, the overall trend indicates a clear correlation between radiation exposure and subsequent cardiovascular events. These observations emphasize the critical importance of long-term cardiovascular monitoring and management strategies for patients who have undergone RT. In the ever-evolving landscape of thoracic oncology, individualized therapeutic decisions should consider the cumulative cardiac risks, weighing the benefits of radiation against the potential cardiovascular sequelae. Collaboration between oncologists, cardiologists, and radiation therapists remains pivotal in optimizing patient outcomes.
